# Supplementary material for: Association between breastfeeding cessation among under six-month-old infants and postpartum depressive symptoms in Nevada
Source: PLoS One. 2024 Jan 26;19(1):e0297218. doi: 10.1371/journal.pone.0297218 (PMC10817202; doi:10.1371/journal.pone.0297218)
Supplement: Attachment — Submitted filename: Plos One-PPD and breasfeeding review.docx [file pone.0297218.s002.docx]

**Manuscript Number:** PONE-D-23-18811
**Manuscript Title:** Association between maternal postpartum depressive symptoms and breastfeeding among six-month-old infants in Nevada.

This well-written article examines maternal postpartum depression and its association with breastfeeding cessation.

Comments:

1. The title needs to be changed/rephrased and this study did not measure among six-month-old infants. Please mention ‘breastfeeding cessation’ and ‘under six months’ in the title.
2. Line 53 – please clarify the term ‘new mother’. Does it refer to first-time mother?
3. Line 86 – statement needs reference/s
4. What are the exclusion criteria? Please describe.
5. As the known prevalence of depression is higher among females, do you include those with mental disorder diagnoses in this study?
6. Line 157 - please explain more about the 24-hour diet recall used to determine breastfeeding practice. Why this method is chosen? Is 24-hour diet recall accurate to measure breastfeeding cessation? If infants are still fed with breast milk a few times a week, does it consider ‘breastfeeding cessation’?
7. Line 167 – please describe WIC
8. Line 196 – Please rearrange the paragraph according to the variables in Table 2. The current arrangement makes it harder to refer to the table.
9. Table 2: Some categories include only very few participants. Even though the result is significant if only three participants are included in the category, how marked the result is? Please kindly discuss this.
10. Relationship between postpartum mental health and breastfeeding and well-versed. What is the difference with your study?
