# Supplementary material for: Association between breastfeeding cessation among under six-month-old infants and postpartum depressive symptoms in Nevada
Source: PLoS One. 2024 Jan 26;19(1):e0297218. doi: 10.1371/journal.pone.0297218 (PMC10817202; doi:10.1371/journal.pone.0297218)
Supplement: Attachment — Submitted filename: 9.11.23-Response to Reviewers.docx [file pone.0297218.s003.docx]

**To the Editor and reviewers of PLOS ONE:**

**Thank you for taking the time to review our manuscript and provide detailed and constructive feedback. We reviewed your comments meticulously and revised them accordingly. We hope they meet your expectations. Please find our point-by-point response below.**

**Reviewer #1**

There is no major issue with the manuscript. It is well written and present everything they intended to present. However, there is no new finding arose from this study. Mental health and breastfeeding practices study are well established and the results supported many other previous studies.

**Response: Thank you for this feedback. While our study did not unveil groundbreaking findings, it contributes to discussing the context of large urban areas in the US, specifically in Nevada where maternal mental health and breastfeeding practices are understudied. We included more about the context in the introduction (page 6, lines 100-109) and in the discussion sections (pages 17-18, lines 258-265).**

1. The title needs to be changed/rephrased and this study did not measure among six-month-old infants. Please mention ‘breastfeeding cessation’ and ‘under six months’ in the title.

**Response: Thank you for specifying this. It has been corrected (page 1, lines 3-5).**

2. Line 53 – please clarify the term ‘new mother’. Does it refer to first-time mother?

**Response: ‘New mother’ refers to mothers who just delivered a baby, either for the first time or not. It has been clarified (page 4, lines 49-52).**

3. Line 86 – statement needs reference/s

**Response: Done. (page 5, lines 89).**

4. What are the exclusion criteria? Please describe.

**Response: Thank you for the comment. We have specified the exclusion criteria (page 8, lines 136-140).**

5. As the known prevalence of depression is higher among females, do you include those with mental disorder diagnoses in this study?

**Response: Mental health diagnoses were not an exclusion criterion. Thus, everyone who was eligible and wished to participate in the survey could do so, irrespective of their mental health status. (page 8, lines 140-142).**

6. Line 157 - please explain more about the 24-hour diet recall used to determine breastfeeding practice. Why this method is chosen? Is 24-hour diet recall accurate to measure breastfeeding cessation? If infants are still fed with breast milk a few times a week, does it consider ‘breastfeeding cessation’?

**Response: As recommended by WHO, we used the 24-hour dietary recall method to assess breastfeeding cessation. We clarified the method used and the definition of the breastfeeding cessation indicator in the methods section (page 9, lines 164-176). In addition, we added a discussion of the potential bias in the discussion section (pages 20-21, lines 328-333).**

7. Line 167 – please describe WIC

**Response: A brief description has been added (page 10, lines 187-190).**

8. Line 196 – Please rearrange the paragraph according to the variables in Table 2. The current arrangement makes it harder to refer to the table.

**Response: Thank you for the suggestion. It has been rearranged. (page 13-14, lines 221-233).**

9. Table 2: Some categories include only very few participants. Even though the result is significant if only three participants are included in the category, how marked the result is? Please kindly discuss this.

**Response: We have rectified the mentioned categories and thoroughly reevaluated the analysis (Table 2, pages 14-15).**

10. Relationship between postpartum mental health and breastfeeding and well-versed. What is the difference with your study?

**Response: Thank you for this feedback. While our study did not unveil groundbreaking findings, it contributes to discussing the context of large urban areas in the US, specifically in Nevada where maternal mental health and breastfeeding practices are understudied. We included more about the context in the introduction (page 6, lines 100-109) and in the discussion sections (pages 17-18, lines 258-265).**

**Reviewer #2:**

The manuscript at hand describes a cross-sectional study on the association between breastfeeding and postnatal depression. The authors conducted an online survey in which 305 mothers living in Nevada (U.S.) participated. After controlling for variables, such as sociodemographic, environmental, and obstetric characteristics, the authors found an association between breastfeeding cessation and postnatal depression. The manuscript is generally of interest, but there are a few concerns that should be addressed, before the manuscript can be accepted for publication.
**Response: Thank you for this feedback. We appreciate your detailed notes to help to improve the manuscript.**

Page 3, line 65 to 66: The authors should state more recent research on the impact of postpartum depression on the development of children. Here, the authors cite Borra et al. (2015). This study is actually not focusing on the association between postpartum depression and infant development.
The authors could cite, e.g.:
Kaplan PS, Danko CM, Everhart KD, et al. Maternal depression and expressive communication in one-year-old infants. Infant Behav Dev. 2014;37(3):398-405.
Murray L, Cooper PJ, Wilson A, Romaniuk H. Controlled trial of the short- and long-term effect of psychological treatment of post-partum depression, 2: impact on the mother-child relationship and child outcome. Br J Psychiatry. 2003;182(5):420-427.
Murray L, Arteche A, Fearon P, Halligan S, Croudace T, Cooper P. The effects of maternal postnatal depression and child sex on academic performance at age 16 years: a developmental approach. J Child Psychol Psychiatry. 2010;51(10):1150-1159.
Schaadt G, Zsido RG, Villringer A, Obrig H, Männel C, Sacher J. Association of Postpartum Maternal Mood With Infant Speech Perception at 2 and 6.5 Months of Age. JAMA Network Open. 2022; 5(9):e2232672.

**Response: Thank you for pointing this out. We added the reference (page 4, line 64).**

Page 3, line 69 a following: The authors state that breastfeeding positively impacts society. The authors should elaborate more on this impact, as it might not be obvious at first sight.

**Response: We restructured the sentence including a new reference (page 4, lines 67-70).**

Page 4, line 74-75: The authors should elaborate a bit more on the statement that the fact that less than half of the world’s infants are being breastfed as recommended, represents a loss of more than $300 billion each year.

**Response: We included new references to support the statement (page 5, lines 72-77).**

Page 4, line 95 to page 5, line 98: I was a little bit confused that authors state that they hypothesized that practicing breastfeeding can positively influence maternal mental health and reduce maternal postpartum depressive symptoms and that their aim was to identify whether maternal postpartum depressive symptoms are associated with early breastfeeding cessation. The hypothesis, from what I understood, is focusing on the direction of breastfeeding leading to less maternal postpartum mood, while the aim is focusing on the other direction that postpartum depression leads to cessation. As the authors are reporting a cross-sectional study, both cannot really be investigated. I strongly encourage the authors to rephrase the last part of the introduction.

**Response: We corrected the hypothesis and rephrased the last part of the introduction (page 6, lines 103-105).**

Page 5, line 101: There seems to be a word missing in this sentence.

**Response: We have corrected the sentence (page 7, lines 112-113).**

Page 8, line 167-168: The word “program” should be omitted once, if I am not mistaken.

**Response: Done (page 10, lines 185-187).**

Page 8, line 179: The authors state that “bivariate analysis between maternal postpartum depressive symptoms was computed across the independent variables”. Here the authors should state why these analyses were performed. At first sight this does not come across.

**Response: We have explained why bivariate analysis was done (page 11, lines 202-206).**

Table 1: I suggest having the same order for “no” and “yes”. For maternal postpartum depression, the authors first report yes-answers, while they first report no-answers for breastfeeding cessation. This should also be checked for the other variables.

**Response: Done. We have checked with other variables and updated the tables accordingly (Table 1, page 12).**

Table 2: I am still not sure whether I understand what the bivariate statistics tell. What exactly did the authors analyze and what does a significant p-value mean. Further, I was confused about the numbers in parenthesis. It is stated that these are percentages. However, the numbers do not add to 100%. Where do the numbers come from? This should be explained or corrected.

**Response: Bivariate analysis is a statistical method to determine if there is a statistical association between the two variables and, if so, how strong and in which direction that association is (reference). Our bivariate analysis is presented in Table 2. First, the cross-tabulation helped to clarify the existence and the direction of an association between maternal postpartum depression outcome, the independent variable, and covariables, individually. For example, among mothers with postpartum depression, 16.25% had ceased breastfeeding compared to 6.67% who did not cease breastfeeding; on the other hand, among mothers without postpartum depression, 83.75% had ceased breastfeeding, while 93.3% did not cease breastfeeding. This description illustrates the direction of a possible association (without any cause-and-effect relationship), which in this case, shows the presence of maternal mental health increases the frequency of breastfeeding cessation. Second, the goal of the chi-square test was to explore how strong the association between maternal postpartum depression and covariables was individual. All covariables with a p-value <0.20 in the bivariate analysis were included in the multivariate logistic regression model.**

**Reference:**

**Bertani, A., Di Paola, G., Russo, E., & Tuzzolino, F. (2018). How to describe bivariate data. *Journal of thoracic disease*, *10*(2), 1133–1137. https://doi.org/10.21037/jtd.2018.01.134**

Discussion: The authors outline in which way their findings are (mainly) inconsistent with other studies. It would be appropriate to also discuss why these inconsistencies might have occurred. I am not familiar with the sociodemographic characteristics of Nevada or the U.S., but to me the sample did not seem to be entirely representative. I was surprised about the proportion of marital status, as well as annual household income. Maybe this could be one of the reasons for inconsistencies? Independent of the reasons, the authors should elaborate on this more carefully.

**Response: Thank you for this note. We added a paragraph discussing the hypothesis of the different findings from our study to other studies as well as how it relates to the Nevada context (pages 19-20, lines 305-317).**

Further, I strongly encourage the authors to discuss more deeply on the causality of their results, especially when considering their aim (see comment above). The authors cannot make any conclusions about causality and therefore cannot conclude that breastfeeding reduces the risk of postpartum depression.

**Response: We agree with your comment. Following the suggestions, we reframed the aim of the study in the background section (page 6, lines 107-109). We added a paragraph expanding on the discussion of the limitations of our study due to sampling and design (page 18, lines 274-279, page 19, lines 300-304, & page 20, lines 318-322). We also redid the conclusion (page 21, lines 335-346).**
