# Supplementary material for: Association between breastfeeding cessation among under six-month-old infants and postpartum depressive symptoms in Nevada
Source: PLoS One. 2024 Jan 26;19(1):e0297218. doi: 10.1371/journal.pone.0297218 (PMC10817202; doi:10.1371/journal.pone.0297218)
Supplement: Attachment — Submitted filename: Response to Reviewers.docx [file pone.0297218.s004.docx]

**To the Editor and reviewers of PLOS ONE:**

**Thank you for taking the time to review our manuscript and provide detailed and constructive feedback. We reviewed your comments meticulously and revised them accordingly. We hope they meet your expectations. Please find our point-by-point response below.**

Reviewer #2: The manuscript at hand is a study I reviewed earlier. The authors have done a good job in revising the manuscript and responding to the reviewer comments.
I have one minor comment left and can then suggest the manuscript for publication.

In my previous comments, I asked the authors to explain why they performed the bivariate statistics. Further, I pointed out that in Table 2, the numbers do not add to 100%. In their revised manuscript the authors explained the aim of the bivariate statistics more precisely and in their response, the authors explained why the numbers in table 2 do not add up to 100%. However, I could not find any information in the manuscript and would strongly suggest adding a note to table 2, to explain the percentage more precisely**.** If I overread this information, please feel free to ignore this comment.

**Response: Thank you for your feedback. We added a footnote in Table 2 explaining 100% is the sum of the numbers in a row (page 14, line 215).**

Reviewer #3: Thank you for the well-written manuscript. However, although I acknowledge that there are no estimates for postpartum depression symptoms, the study was conducted with a small sample using purposive sampling and a cross-sectional study design, so this manuscript does not contribute new knowledge to the field.

**Response: Thank you for the opportunity to reflect on your contribution to new knowledge in the field. Our team believes that research should always seek to strike a balance between (1) context-specific knowledge as well as (2) transferability. Our study contributes to the field by being the first to report the prevalence of postpartum depressive symptoms and investigate the independent association with breastfeeding cessation in Nevada (context). Furthermore, while corroborating existing literature, it also invites readers of the research to make connections between elements of our study and their own experiences (transferability). Collectively, our findings contribute to context-specific hypotheses and support transferability for future public health research exploring the relationship between postpartum depression and breastfeeding. Therefore, we believe that this manuscript, despite its limitations (detailed in the discussion section), makes a valuable contribution to the literature and the field of public health.**

Reviewer #4: You provide these two statements: Line 27 (abstract): “We aimed to analyze whether maternal postpartum depression symptoms are associated with early breastfeeding cessation”; Line 108 (background): “Thus, we aimed to find out whether early breastfeeding cessation is an independent risk factor for maternal postpartum depression”. You should be clearer about the aim of the study/ in which direction are you seeking an association.

**Response: Thank you for pointing this out. We now have stated our aim in a consistent way or direction: ‘To analyze whether breastfeeding cessation is associated with postpartum depression among mothers of infants under six months old in Clark County, Nevada (both in title, abstract (lines 26–27), and introduction (lines 91–92)).**

Line 171: A 24h dietary recall is not the best method, since a mother could need to transiently interrupt breastfeeding due to medication/medical exams and resume again later. You explain this in the discussion, however, if a mother was not breastfeeding, how do you know when she stopped? If you don’t have this information, you should state it as a limitation of your study.

**Response: Thank you for this comment. Specifically, we do not have information on when the mother stopped breastfeeding and have included it in the limitation paragraph. While we acknowledge the potential limitations of a 24-hour dietary recall, we used it to standardize measurement when assessing infant feeding outcomes and to avoid recall bias as recommended by the WHO. It also allows future comparisons across studies and populations. We added a paragraph in the discussion section to discuss the limitations pointed out by the reviewer (Page 18, lines 304–309).**

Besides, did you have the option “breastmilk + formula” in your questionnaire?

**Response: We did ask about mixed feeding with ‘breastmilk+formula’ in the 24-hour dietary recall. Because our independent variable refers to any breastfeeding, in the case of the affirmative response, this dyad was classified as breastfeeding (Page 7, lines 154-157).**

Line 177: Regarding the covariables, did you ask the mothers if they had a diagnosis of depression/other psychiatric disorder and/or if they were taking any medication? It is important to know this as some treatments are not compatible with breastfeeding.

**Response: We do acknowledge the importance of these questions; however, unfortunately, they were not included in the questionnaire. We added a paragraph in the discussion section to discuss the limitations pointed out by the reviewer (Page 18, lines 307-309).**

Table 1: I suggest you write “postpartum depression symptoms” instead of “maternal postpartum depression” in your table, since you do not have a clinical diagnosis.

**Response: We standardize “postpartum depressive symptoms” throughout the manuscript. It also has been corrected in Table 1.**

Table 1: There is a high prevalence of newborns separated from their mothers in the first hour, yet a low prevalence of admissions in the neonatal unit. While it is not the primary goal of the study, this finding deserves some discussion.

**Response: Thank you for this feedback. We added a paragraph to discuss this important insight in our discussion (Page 16, lines 259 - 269).**

Table 2: You should not write in the title “risk for” since you did not evaluate causality.

**Response: Thank you for your suggestion. We removed it throughout the manuscript.**

Line 305: there is a grammatical error.

**Response: Thank you for noticing this. It has been corrected (Pages 20, line 312-316).**

Overall, the study is well written and gives information about the prevalence of postpartum depression symptoms and mothers who did not breastfeed in the previous 24h. While this is useful information and the study has a good sample size, there are some variables that were not taken into account: there is no information regarding a diagnosis of depression or medication taken by mothers, no information about the timing of breastfeeding cessation or if there were mothers who never breastfed. These are important limitations and should be mentioned.

**Response: Thank you for your feedback. We updated this information in our discussion, where we specified our limitations (Page 18, lines 304-309).**
